# Comparative study on physicochemical properties, microbial composition, and the volatile component of different light flavor *Daqu*

**DOI:** 10.1002/fsn3.3476

**Published:** 2023-06-12

**Authors:** Panpan Hu, Ji Wang, Urooj Ali, Tariq Aziz, Manal Y. Sameeh, Caiping Feng

**Affiliations:** ^1^ Department of Life Science Lyuliang University Lyuliang Shanxi China; ^2^ College of Food Science and Engineering Shanxi Agricultural University Jinzhong Shanxi China; ^3^ Department of Biotechnology Quaid‐i‐Azam University Islamabad Pakistan; ^4^ School of Food and Biological Engineering Jiangsu University Zhenjiang Jiangsu China; ^5^ Chemistry Department, Faculty of Applied Sciences, Al‐Leith University College Umm Al‐Qura University Mecca Saudi Arabia

**Keywords:** *Daqu*, light flavor, microbial community structure, physicochemical properties, volatile components

## Abstract

Baijiu, a type of liquor, is known for its pure fragrance and softness. Its unique style is attributed to the complex microbial flora and flavor precursors found in *Daqu*. In order to elaborate the nature of light flavor *Daqu* to guide the baijiu production, four *Daqu* samples (*DQ1*, *DQ2*, *DQ3*, and *DQ4*) from Shanxi province were analyzed to determine their microbial structure, physicochemical properties, and volatile flavors using high‐throughout put seqencing and headspace solid‐phase microextraction/gas chromatography–mass spectrometry method in this study. The findings indicated that there were no noticeable variations in the water content and esterase activity of the four *Daqu*. However, the DQ2 sample had a higher acidity value and saccharifying enzyme activity, whereas DQ3 had the highest protease activity. The microbial community structure of the four *Daqu* was similar, with *Lactobacillus* and *Streptophyta* as the dominant bacteria, but the abundance of bacteria was different among the four *Daqu*. *Issachenkia* was a common dominant fungus genus in all samples. *Rhizopus* and *Lichtemia* were higher in DQ1 and DQ2, while *Torulaspora*, *Aspergillus*, and *Candida* were more prevalent in DQ4. A total of 27 volatile components were detected in the four *Daqu*, including esters, alcohols, ketones, aldehydes, and acids. DQ2 had the most volatile components and ethyl lactate and ethyl acetate were the most significant esters in the four samples. In conclusion, the physicochemical indicators of the four light flavor *Daqu* had distinct differences. There were significant variations in the abundance of bacteria and fungi, leading to differences in the volatile component content. These research findings can serve as a theoretical foundation for blending different light flavors *Daqu* and hold great significance in enhancing the quality of baijiu.

## Graphical abstract

The physicochemical indicators of the four light flavor Daqu had distinct differences. There were significant variations in the abundance of bacteria and fungi, leading to differences in the volatile component content. These research findings can serve as a theoretical foundation for blending different light flavor Daqu and hold great significance in enhancing the quality of baijiu.

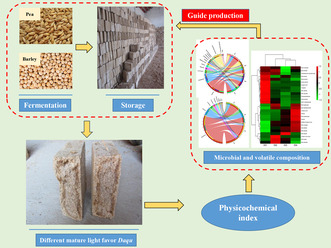

## 1 INTRODUCTION

As one of China's ancient and distinctive alcoholic drinks, baijiu is famous worldwide (Wu et al., 2021). It is made with grains as raw materials (Hu et al., 2023) and *Daqu*, *Xiaoqu*, or *Fuqu* as saccharifying starter cultures after steaming, saccharifying, fermentation, distillation, and other processes (Yang et al., 2017). Due to the different raw materials, production technology, and blending technology used in the brewing process, the baijiu finally presents different style characteristics and the feeling of the entrance is also not the same, thus forming unique flavors, and baijiu is further categorized into different types: Light flavor baijiu, Nongxiang flavor baijiu, Maotai flavor baijiu, and rice flavor baijiu (Zhang et al., 2014).

*Daqu*, as a saccharifying starter containing microorganisms and enzymes, affects the style and yield of baijiu (Jin et al., 2019). In actual production, different types of *Daqu* are often mixed according to the season, raw materials, fermentation characteristics, etc., to ensure the quality of baijiu (Wu et al., 2020). According to the different production temperatures, *Daqu* can be classified into high‐temperature (60–65°C), medium‐temperature (50–60°C), and low‐temperature *Daqu* (40–50°C) (Cai et al., 2022). The *Daqu* used for light flavor baijiu is a medium‐low temperature koji with barley and peas as the primary raw materials (Liu et al., 2019). The temperature of *Daqu* making is 45–50°C, which is suitable for the growth and propagation of various microorganisms. The fermented metabolites of these microorganisms, such as acids, aldehydes, and esters, affect the quality and flavor of baijiu (Tian et al., 2020; Xie et al., 2020).

In the production of light flavor, baijiu, *Hongxin Daqu*, *Houhuo Daqu*, and *Qingcha Daqu* are usually mixed for production (Ling et al., 2020). Microorganisms in these *Daqu* are naturally enriched in the open production process (Wang, Ban, Hu, et al., 2017). It contains a variety of microorganisms, including bacteria, mold, and yeast (Mao et al., 2022). Yeast plays a decisive role in the fermentation process of baijiu. In the brewing process, yeast can use nutrients to produce ethanol and carbon dioxide (Zheng et al., 2012) and form some flavor compounds, including alcohol, sulfur, nitrogen, esters, volatile and phenols, lactones, furans, and other substances (Hu et al., 2021). These flavor compounds make essential contributions to the aroma and taste of baijiu. In addition to yeast, other microorganisms such as molds can produce gluconase, alpha‐amylase, carboxypeptidase, and acid protease (Li et al., 2013), which can generate flavor compounds by hydrolyzing nutrients such as starch and protein in raw materials (Guo et al., 2020). *Lactic acid bacteria* (LAB), *Streptococcus*, and other bacteria can secrete protease to hydrolyze starch and other raw materials to produce pyrazine and alcohol substances, promoting the Maillard reaction and producing particular flavor substances of baijiu (Xiao et al., 2017).

At the same time, these bacteria can produce lipase, increasing the esters' content. They are conducive to shortening the wine's aging time and making its aroma fuller and softer (Wang, Sun, et al., 2020; Wang, Wang, et al., 2022). Shanxi Province is the largest producer of light flavor baijiu in China. Compared with other *Daqu*, light flavor *Daqu* has a lower temperature in the making process, so the microbial species are more abundant (Zhang et al., 2012). At present, many studies have introduced the microbiological composition and physicochemical properties of common *Daqu* in detail, including Maotai flavor *Daqu* (Xiao et al., 2017), Jiangxiang flavor *Daqu* (Cai et al., 2022), etc., but there are few reports on the light flavor *Daqu*. Meanwhile, most studies only study the volatile components of baijiu, and few studies on the composition of volatile substances in *Daqu*. Therefore, it is significant to study the microbial composition and volatile components of the light flavor *Daqu* to improve baijiu's quality.

The microbial composition and diversity of Daqu vary depending on the flavor and type of baijiu and the geographical region of production. However, mixing different types of Daqu in baijiu production is often based on empirical knowledge, lacking a strong scientific basis. Therefore, this study utilizes advanced technologies including high‐throughput sequencing technology (NGS) and headspace solid‐phase microextraction/gas chromatography–mass spectrometry (HS‐SPME–GC–MS) to investigate the microbial community structure and volatile components of light flavor Daqu from various regions in Shanxi province. Additionally, the physicochemical properties of Daqu are analyzed to provide a more comprehensive understanding of the potential for mixed‐use of different types of Daqu in baijiu production. These findings provide a theoretical basis for improving the quality and production of light flavor baijiu.

## 2 MATERIALS AND METHODS

### 2.1 Sample collection

Samples of four light‐flavor Daqu were collected from Xiaoyi, Fenyang, Taiyuan, and Jiaocheng in Shanxi Province, China. The *Daqu* samples were composed of a mixture of Hongxin *Daqu*, Houhuo *Daqu*, and Qingcha *Daqu*, labeled as DQ1, DQ2, DQ3, and DQ4. Samples were collected in April spring. The above samples were broken and mixed directly by pulverizer (Henan Xinda), divided into aseptic bags for packaging and marking, and transported back to the laboratory under 4°C condition. Then, screened through a 40‐mesh sieve and stored at −80°C refrigerator (Thermo FisherScientific) for future use, and follow‐up experiments were shown in the graphical abstract.

### 2.2 Determination of physicochemical indexes of *Daqu*

Moisture, acidity, esterification ability, saccharification ability, and protease activity of *Daqu* samples were measured by referring to the method of Fan et al. (2015). The moisture (%) is expressed as a percentage. Acidity (mmol/10 g) is expressed as the millimole number of 0.1 mol/L sodium hydroxide standard solution consumed by 10 g of absolute dry *Daqu*. Saccharification ability (U/g) represents the amount of 1 g dried *Daqu* converted into glucose in 1 h under the condition of 35°C and pH = 4.6. Esterification ability (mg/g) represents the amount of ethyl caproate synthesis catalyzed by caproic acid and ethanol at 35°C for 7 days per 50 g *Daqu*. Protease activity (U/g) refers to the amount of tyrosine produced by hydrolyzing casein with 1 g *Daqu* per minute under the condition of 40°C.

### 2.3 Analysis of microbial diversity in *Daqu* samples

The samples (7 g) were washed with 10 mL of 0.1 mol/L phosphate‐buffered solution to collect bacteria. The microbial genome of Daqu was extracted using the Omega EZNATM. soil genome DNA extraction kit. For PCR amplification of bacterial 16S rRNA V3‐V4 region and fungal ITS1‐ITS2 region, primers 338F/806R (5′‐GTGYCAGCMGCCGCGGTAA‐3′/5′‐GGACTACNVGGGTWTCTAAT‐3′) and ITS1F/ITS2R (5′‐CTTGGTCATTTAGAGGAAGTAA‐3′/5′‐GCTGCGTTCTTCATCGATGC‐3′) were used, respectively. PCR amplification products were then subjected to 2% agarose gel electrophoresis and recovered by gelatinizing with an AxyPrep DNA gel recovery kit. Shanghai Sangong Bioengineering Co., Ltd. was commissioned for high‐throughput sequencing of the purified PCR amplification products. After splicing using FLASH (1.2.11), operational taxonomic units (OTU) were divided with Usearch (10) at 97% similarity, and the annotations were compared with Silva (138.1) and Unite (8.2) databases.

### 2.4 Analysis of volatile substances in *Daqu* by HS‐SPME–GC–MS

*Daqu* sample (1 g) was mixed with 10 μL 2‐octanol (10 mg/L) of internal standard in headspace vials. SPME fiber (50:30 mm divinylbenzene‐carboxy‐polydimethylsiloxane) was used to extract at 60°C for 30 min. Compounds were separated on the DB‐Wax column (30.0 m × 0.25 mm × 0.25 μm). Gas chromatographic conditions: Helium was used as carrier gas at a constant flow rate of 0.8 mL/min. Heating procedure: initial temperature was 40°C maintained for 5 min, 6°C/min went up to 100°C, and then 10°C/min went up to 230°C. The electron energy was set to 70 eV, and the scanning range was 33–400 m/z. The M.S. source and four‐stage rod temperature were 200 and 230°C, respectively. Each compound was identified using the National Institute of Standards and Technology (NIST) database. The relative concentration of *Daqu* volatile flavor was calculated according to the ratio of the internal standard peak area to the flavor substance peak area.

### 2.5 Statistical analysis

The richness of OTUs was measured using Chao1, Simpson, and Shannon indices to determine the α‐diversity indices. Rarefaction curves of observed OTUs were created to evaluate the sample adequacy, and Mothur software (version 3.8.31) was used to calculate all α‐diversity indices. OTU rarefaction and rank abundance curves were generated in R (version 3.6.0). The *T*‐test was performed to calculate the within‐sample (alpha) diversity for two groups, and ANOVA was used for multiple group comparisons to estimate the diversity of the microbial community. To visualize differences in the microbiome among samples, beta diversity was evaluated and combined with constrained principal component analysis (PCA). These analyses were visualized using the vegan package (version 2.5‐6) in R, and inter‐sample distances were presented as scatterplots. STAMP (version 2.1.3) and LefSe (version 1.1.0) were used for difference comparison to identify features with significantly different abundances between groups.

## 3 RESULTS AND DISCUSSION

### 3.1 Determination of physicochemical properties of *Daqu*

The physicochemical properties of *Daqu* can be compared using indicators such as moisture content, acidity, protease, esterase, and saccharifying enzyme (Moreno et al., 2006). Table 1 shows no significant difference in moisture content among the four *Daqu* samples (*p* > .05). The moisture content indicates the amount of water contained in *Daqu*. With the extension of storage time, the moisture content of *Daqu* gradually decreases, indicating that the more free water produced by fermentation, the stronger the volatilization ability and the better the maturity of *Daqu*. According to the national standards for *Daqu*, its moisture content cannot exceed 13% of the total mass (Zheng et al., 2017), and four *Daqu* samples are qualified. *Daqu* acidity is an important indicator that reflects the metabolism of *Daqu* microorganisms and assesses the quality of *Daqu*. During the production of light flavor *Daqu*, a complex fermentation process occurs, and the acidity of *Daqu* is precisely produced by the organic metabolism of acid‐producing microorganisms (Hu et al., 2012). The results show that the maximum acidity value of DQ1 is 0.960.96 ± 0.21 mmol/10 g, significantly different from DQ1, DQ2, and DQ3 (*p* < .05).

**TABLE 1:** Physicochemical indexes of *Daqu*.

	DQ1	DQ2	DQ3	DQ4
Moisture (%)	6.36 ± 0.32^a^	7.13 ± 0.53^a^	7.33 ± 0.23^a^	6.34 ± 0.34^a^
Acidity (mmol/10 g)	0.75 ± 0.13^b^	0.96 ± 0.21^a^	0.68 ± 0.19^b^	0.71 ± 0.27^b^
Saccharification ability (U/g)	457.67 ± 7.45^c^	503.32 ± 8.64^a^	486.33 ± 4.87^b^	498.55 ± 7.11^b^
Protease activity (U/g)	104.33 ± 2.67^bc^	89.66 ± 1.87^c^	166.67 ± 2.77^a^	119.55 ± 2.44^b^
Esterification ability (mg/g)	553.43 ± 9.65^a^	598.55 ± 7.71^a^	587.66 ± 6.22^a^	573.22 ± 6.45^a^

*Note*: The exact line marked with different lowercase letters indicates a significant difference (*p* < .05).

The enzyme system in *Daqu* is the power to decompose raw materials such as starch and protein in *Daqu*, and the decomposition products provide a material basis for microbial metabolism. Aspergillus, Mucor, Rhizopus, and Bacillus are widely present in *Daqu*, and the growth and reproduction status of these proteases produced by microorganisms is a critical factor in the level of protease activity in *Daqu* (Xu et al., 2013). Proteinase can decompose proteins to generate amino acids, which can participate in the Maillard reaction to generate flavor substances, promoting flavor precursors' formation in *Daqu*. From the results, it can be seen that the maximum protease activity was 166.67 ± 2.77 U/g for the DQ3 sample, which was significantly higher than that of the DQ4 sample (*p* < .05), and the lowest protease activity was 89.66 ± 1.87 U/g for DQ2 sample, which was not significantly different from DQ1 sample (*p* > .05), but significantly different from DQ4 (*p* < .05).

Although there is no significant difference in the esterase activity of each *Daqu* sample, there is a significant difference in the saccharifying enzyme activity of the four samples. The maximum saccharifying enzyme activity of the DQ2 sample was 503.32 ± 8.64 U/g, significantly higher than that of the DQ3 and DQ4 samples. The minimum saccharifying enzyme activity of the DQ1 sample was 457.67 ± 7.45 U/g, significantly different from that of the DQ3 and DQ4 samples (*p* < .05). Ester compounds and alcohol compounds are closely related to the formation of *Daqu* flavor. The esterase and saccharifying enzyme activities of *Daqu* are the driving force for the catalytic production of these compounds. The higher Daqu produces the enzyme activity, the more ester and alcohol compounds during fermentation, which is conducive to promoting the formation of the baijiu flavor (Zhang et al., 2012). Like our results, Fu et al. (2021) examined the physical and chemical properties of *Daqu* in different seasons. The results showed that the esterifying enzyme activity and liquefaction enzyme activity of *Daqu* in summer were significantly greater than those of *Daqu* samples in autumn, thus indicating that the production of *Daqu* can be affected by various factors such as season, temperature, and humidity leading to significant differences in the final physical and chemical properties, as concluded in our study.

### 3.2 Analysis of basic sequence statistics

The extracted PCR products were sequenced using the Illumina Miseq sequencer, and effective sequences were obtained using the double‐terminal sequencing method. Finally, the primary sequence information of four *Daqu* samples was obtained. Moreover, the detailed results are shown in Table 2.

**TABLE 2:** The basic sequence information of sample sequencing.

	Sample	SeqNum	BaseNum	MeanLen	MinLen	MaxLen
Bacteria	DQ1	66,892	28,158,812	420.96	350	474
	DQ2	67,816	28,337,881	417.86	353	471
	DQ3	87,441	36,647,612	419.11	365	448
	DQ4	84,068	35,609,417	423.58	357	468
	Total	306,217	128,753,722			
Fungi	DQ1	79,198	16,861,109	212.9	101	437
	DQ2	60,963	15,440,190	253.27	101	449
	DQ3	131,058	24,893,410	189.94	110	441
	DQ4	99,631	20,127,851	202.02	110	441
	Total	370,850	77,322,560			

It can be seen that in the bacterial sequencing results, the optimized sequence number reaches 2 × 306,217, among which the adequate sequence number of the DQ1 sample is 2 × 66,892, the valid sequence number of the DQ2 sample is 2 × 67,816, the adequate sequence number of DQ3 sample is 2 × 87,441, the correct sequence of DQ4 sample is 2 × 84,068. The total number of effective base pairs of bacteria was 2 × 128,753,722 bp, among which the most significant number in the DQ3 sample was 2 × 36,647,612 bp, and the least number of base pairs in DQ1 was 2 × 2,815,882 bp. In the results of fungal sequencing, a total of 2 × 370,850 bp was obtained by optimizing each sample sequence, in which the adequate sequence number of the DQ1 sample was 2 × 79,198 bp, that of the DQ2 sample was 2 × 60,963 bp, and that of DQ3 sample was 2 × 131,058 bp. The effective sequence of the DQ4 sample was 2 × 99,631 bp. After removing joint pollution, low complexity, and low‐quality base pairs, the total fungal base was 2 × 77,322,560 bp, in which the number of base pairs in DQ3 was the largest, reaching 2 × 20,127,851 bp, and the number of base pairs in DQ2 was the least. Up to 2 × 15,440,190 bp, the unworded sequence was the target sequence, providing reliable data for future bacterial diversity analysis.

### 3.3 Rarefaction curve and rank‐abundance curve

The high‐throughput sequencing results were evaluated to make the rarefaction and rank‐abundance curves for bacteria and fungi. The rarefaction curve is mainly used to reflect the richness of species and the diversity of microbial colony structures contained in different samples. The trend of the rarefaction curve can provide a good understanding of whether the sequencing data amount is reasonable to provide a basis for future analysis (Fan et al., 2019). The rarefaction curve's smoothness can reflect the sequencing data adequacy. When the curve is at the rising stage, it indicates that the sample sequencing depth is insufficient and more data are needed. When the curve tends to flatten, it indicates that the measured data can reflect all species information of the sample. There is no need to increase the amount of data, as adding more data can only generate a small amount of new OUT. The rank‐abundance curve can visually show the abundance and evenness of species in each sample. In the horizontal direction, species abundance is ultimately reflected in the curve's width. The greater the width of the curve on the abscissa axis, the greater the species variety. The smoothness of the curve reflects the homogeneity of the species in the sample; the smoother the curve, the more uniform the species distribution is (He et al., 2020).

**FIGURE 1:**
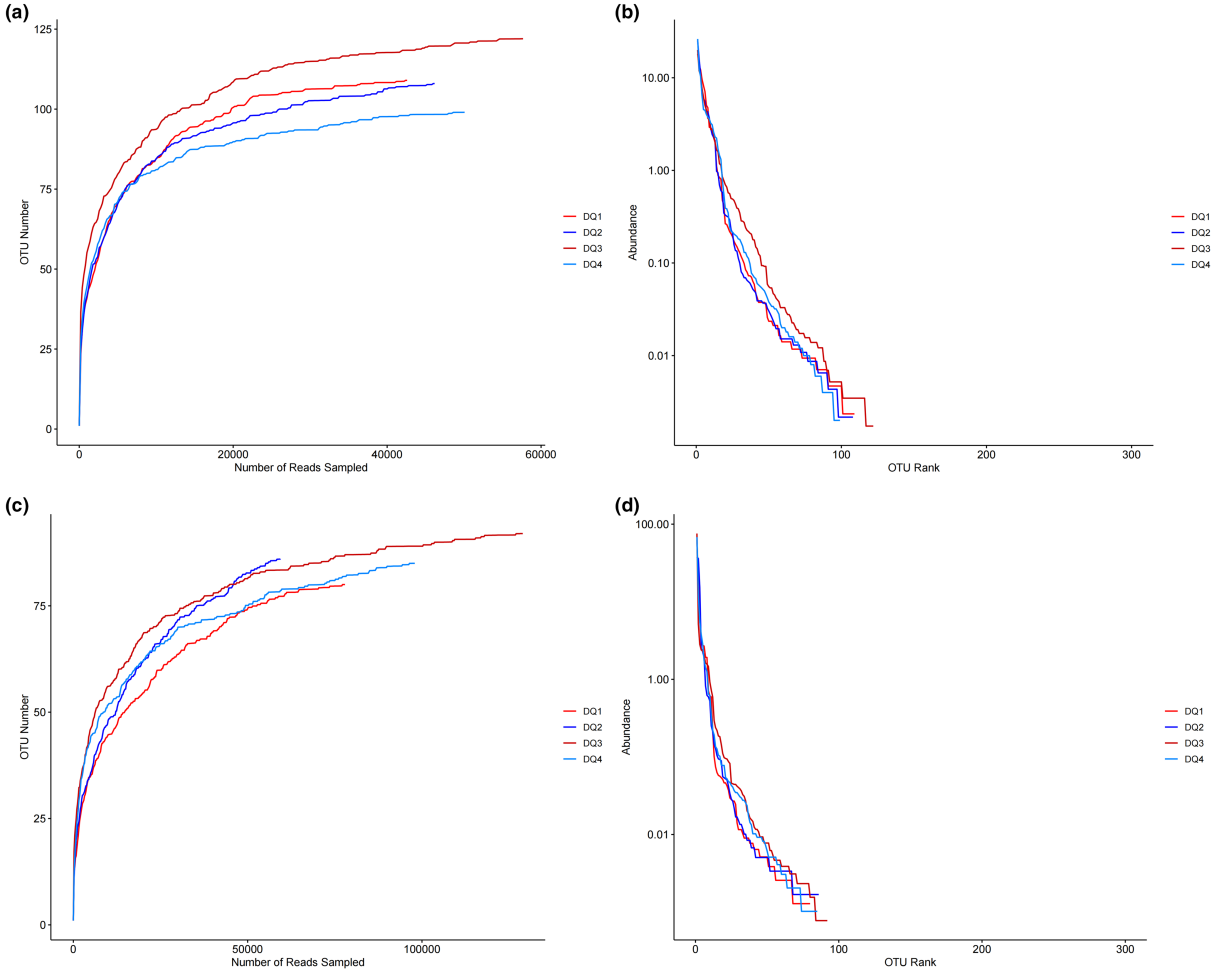
Evaluation of sequencing results. (a) Rarefaction curve of bacteria; (b) rank‐abundance curve of bacteria; (c) rarefaction curve of fungi; (d) rank‐abundance curve of fungi.

From the results in Figure 1a,c, it can be seen that the bacterial and fungal rarefaction curves of the four *Daqu* samples have tended to be flat, indicating that the sequencing depth of the four *Daqu* samples is reasonable, and the sample number obtained can also contain all bacterial information. At the same time, it also indicates that all samples contain rich bacterial species and a high degree of bacterial diversity, which is of particular significance. As can be seen from Figure 1b, the bacterial rank‐abundance curve of the four samples is steep and wide, indicating that the distribution of fungi in the four samples varies greatly, with high abundance and rich sample information. Figure 1d shows that the fungal rank‐abundance of the four Daqu samples decreased rapidly in the vertical direction. The curve was relatively steep, indicating the abundance of fungi in different *Daqu* samples was relatively low, and there were noticeable differences.

### 3.4 α‐Diversity analysis

The α‐diversity analysis can reveal the species richness and diversity of the microbial community in the sample (He et al., 2019). ACE, Chao, Shannon, and Simpson indexes are generally used in the analysis. As shown in Table 2, the Shannon index and Simpson index represent the diversity of species in the sample. The larger the Shannon value, the higher the species diversity in the community. On the contrary, the larger the Simpson index value, the lower the species diversity in the community (Jeon et al., 2016). The ACE and Chao indexes represent species richness, while the coverage index represents the size of species coverage in the sequencing results (Hong et al., 2019).

The α‐diversity analysis was performed with the noise‐reduced sequences. The diversity indices of prokaryotic and eukaryotic microorganisms were obtained (Table 3). The coverage rate was above 99.9%, indicating that the sequencing results could cover the microbial communities in all samples, which were more representative and could genuinely reflect the diversity and abundance of species in the samples (Wang, Wu, et al., 2019). The species diversity and abundance of prokaryotic microorganisms in all samples were generally higher than those of fungal species. According to the results, 196,491 bacterial sequences were detected in the four *Daqu* samples.

**TABLE 3:** α‐Diversity indexes of *Daqu*.

	Sample	Number	Abundance indexes			Diversity indexes		
			Chao1	ACE	Coverage	Shannon	Simpson	Coverage
Bacteria	DQ1	42,598	112.27	112.92	0.999	0.58	0.10	0.999
	DQ2	46,169	114.88	112.95	0.999	0.56	0.12	0.999
	DQ3	57,657	122.88	125.95	0.999	0.62	0.09	0.999
	DQ4	50,067	100.11	101.07	0.999	0.60	0.11	0.999
Fungi	DQ1	77,912	86.00	88.26	0.999	0.32	0.42	0.999
	DQ2	59,424	96.06	103.47	0.999	0.34	0.30	0.999
	DQ3	128,905	99.20	96.46	0.999	0.27	0.27	0.999
	DQ4	97,938	91.00	93.72	0.999	0.30	0.30	0.999

The Shannon index, Chao1 index, and ACE index of DQ3 were the highest, while the Simpson index was the lowest, indicating that its community had the highest species richness and diversity. The Shannon index, Chao1 index, and ACE index of the DQ4 sample are the smallest, while the Simpson index is the largest, indicating that its species richness and diversity are the lowest. The species diversity and richness of bacterial communities in DQ1 and DQ2 samples were between DQ3 and DQ4. A total of 364,179 fungal sequences were detected in four *Daqu* samples, with the highest Shannon index, Chao1 index, and ACE index in DQ3 samples, indicating that their community has the highest species richness and diversity.

The Shannon index, Chao1 index, and ACE index of the DQ1 sample are the smallest, while the Simpson index is the largest, indicating that its species richness and diversity are the lowest. The species diversity and richness of bacterial communities in the DQ2 and DQ4 samples were between the DQ3 and DQ4 samples.

### 3.5 Venn analysis

The Venn diagram is mainly used to represent the distribution of OTUs in each sample and can show the similarity and commonality of OTU composition of each sample in detail.

**FIGURE 2:**
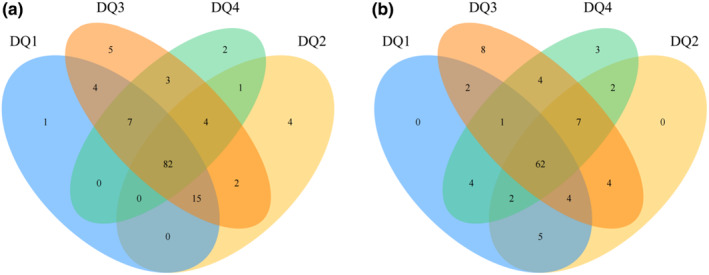
Vene analysis of species in the four *Daqu*. (a) Bacteria; (b) fungi.

As can be seen from Figure 2a, the number of bacterial OTUs in sample DQ1 was 109, the number of bacterial OTUs in sample DQ2 was 108, the highest number of bacterial OTUs was 122 for DQ3 and the lowest number of bacterial OTUs was 99 for DQ4. The number of OTUs in the four samples was relatively similar. The total number of OTUs of bacteria in the four *Daqu* samples was 82, most of which were Actinobacteria, Bacteroides, Cyanobacteria, Elusimicrobia, Firmicutes, and Proteobacteria. As can be seen from Figure 2b, there were 80, 86, 92, and 85 fungal OUTs in the DQ1, DQ2, DQ3, and DQ4 samples, respectively, with no significant difference in the number of OUTs among the four samples. It was consistent with the data of the α‐diversity index of the microbial community in Table 2, with a maximum OUT of 92 in the DQ3 sample, indicating that its community has the highest species richness and diversity. The minimum OUT value of the DQ1 is 80, indicating that its species community has the lowest richness and diversity. There were 62 OTUs in the four *Daqu* samples, most of which were Ascomycota, Basidiomycota, and Mucoromycota.

### 3.6 Analysis of microbial community structure in *Daqu*

**FIGURE 3:**
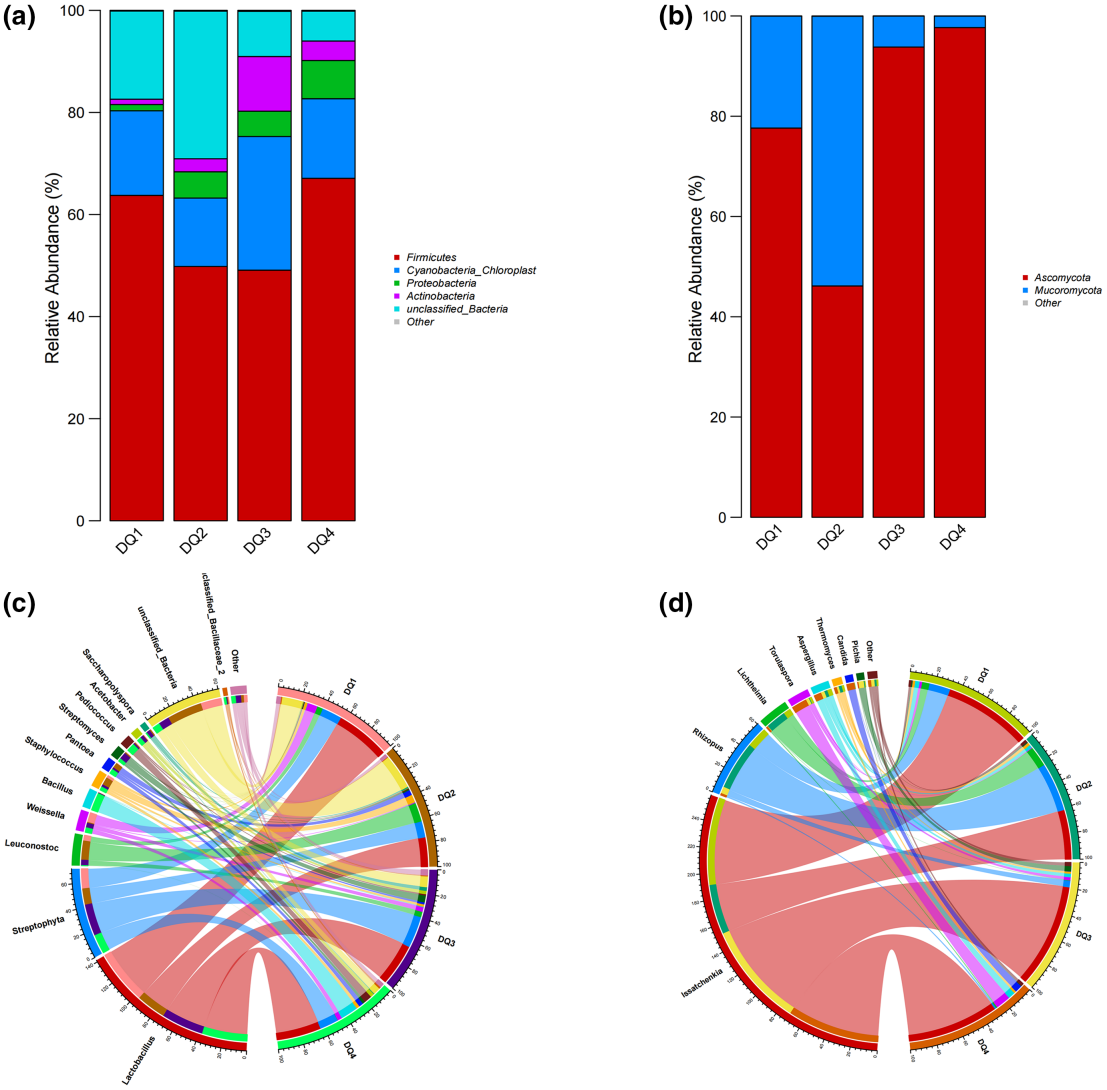
Analysis of the microbial community structure of the four *Daqu* samples. (a) Phylum level of dominant bacteria; (b) phylum level of dominant fungi; (c) genus level of dominant bacteria; (d) genus level of dominant fungi.

To understand the composition of the microbial flora of the four types of *Daqu*, the OTU representative sequence of the bacterial flora was compared and annotated with the homology of the Silva database. Figure 3a shows that the four samples' dominant bacteria at the bacteriological level were *Firmicutes*, *Cyanobacteria*, *Proteobacteria*, and *Actinobacteria*, and the bacterial flora structure of the four *Daqu* samples was different. The abundance of *Firmicutes* was highest in DQ4 and lowest in DQ3 (67.09% and 49.09%, respectively). The abundance of *Cyanobacteria* was the highest in DQ3 and the lowest in DQ2 (26.18% and 13.40%, respectively). The abundance of *Proteobacteria* was the highest in DQ4 and the lowest in DQ1 (7.47% and 1.22%, respectively). The abundance of *Actinobacteria* was the highest in DQ2 and the lowest in DQ4, which were 28.93% and 5.86%, respectively.

As shown in Figure 3c, the dominant bacteria in the four samples were *Lactobacillus*, *Streptophyta*, *Leuconostoc*, *Weissella*, *Bacillus*, *Staphylococcus*, *Pantoea*, *Streptomyces*, *Pediococcus*, *Acetobacter*, and *Saccharopolyspora*. The abundance values of different *Daqu* bacterial genera differed significantly. *Lactobacillus* and *Weissella* had the most considerable abundance in DQ1 samples, 45.12% and 8.27%, respectively. *Leuconostoc*, *Streptomyces*, and *Streptomyces* had the highest abundance in DQ2 samples, which were 15.76%, 6.48%, and 4.36%, respectively. The abundance of *Streptococcus*, *Staphylococcus*, and *Saccharopolyspora* was the highest in the DQ3 samples, with 26.17%, 5.72%, and 2.98%, respectively. *Bacillus*, *Pediococcus*, and *Acetobacter* abundance was the highest among the DQ4 samples, with 14.45%, 6.44%, and 3.10%, respectively.

Studies have shown differences in the microbial composition of *Daqu* produced in different regions and production methods, and the bacteria in *Daqu* mainly come from starter‐making materials (Huang et al., 2017). LAB are the dominant bacteria in *Daqu*, which can promote the Maillard reaction and maintain the microecological environment in baijiu fermentation. LAB is also one of the promoters of the succession of baijiu flavor substances (McClendon et al., 2012). During fermentation, *Lactobacillus* can produce organic acids, flavor substances, and extracellular polysaccharides (Pang et al., 2018). *Weisslla* and *Pediococcus* can hetero‐typically ferment glucose to produce lactic acid through hexose phosphate and phosphoketolase pathways. They can also ferment sugars such as maltose and sucrose to produce acid to increase organic acids, short‐chain fatty acids, esters, and other flavor substances in foods while preventing spoilage (Wang et al., 2018).

*Acetobacter* can produce acetic acid during fermentation, vital in producing fermentation precursors of light flavor baijiu. *Saccharopolyspora* is commonly found in light flavor *Daqu* and has various catalytic hydrolysis functions. *Staphylococcus* exists in the starter‐making environment, *Daqu* and fermented grains, and can produce lipase. Lipase metabolites can form baijiu flavor substances or precursors (Perry et al., 2017). Bacillus can also secrete and ferment amylase to produce pyrazine, an aromatic flavor substance. Wang, Li, et al. (2022) determined the microbial community structure of Luzhou‐flavor baijiu in four different regions of Henan province. The results showed that the dominant bacteria in *Daqu* included *Lactobacillus*, *Weissella*, and *Pediococcus*. It was similar to our results, indicating that these bacteria all play an essential role in the fermentation process of a different flavor of baijiu.

The fungi with an abundance ratio of less than 1% in all samples were classified as Other. The horizontal dominant bacteria diagram of the phylum fungi was obtained, as shown in Figure 3b. The four samples' dominant bacteria at the phylum Fungi level were *Ascomycota* and *Mucormycota*, respectively. There were differences in microbial abundance at the phylum level among different *Daqu* samples. The highest average abundance of *Ascomycota* was 97.68% in DQ4, and the lowest was 46.14% in DQ2. The highest abundance of *Mucormycota* in DQ2 was 53.80%, and the lowest in DQ4 was 2.28%. There were also specific differences in the level of fungal genera among the four *Daqu* samples. As shown in Figure 3d, the highest abundance of the genus *Issatchenkia* in DQ3 was 80.99%, and the lowest in DQ2 was 38.65. The highest abundance of *Rhizopus* in DQ2 was 37.86%, and the lowest in DQ4 was 1.26%. In addition, the highest abundance of *Lichtheimia* in DQ2 was 15.85%. *Torulaspora*, *Aspergillus*, and *Candida* had the highest abundance in DQ4, with abundance values of 11.63%, 5.44%, and 5.64%, respectively, and the highest abundance of *Pichia* in DQ3 was 4.09. There was no significant difference in the abundance values of *Thermomycetes* among the four samples.

Wang, Ban, and Qiu (2017) found that *Rhizopus*, *Lichtheimia*, and *Aspergillus* can produce abundant glucose amylase, which can directly degrade starch in raw materials into reducing sugar available to yeast, thus stimulating yeast growth. At the same time, it also promoted the conversion of carbon sources from raw materials to alcohol, which was the final product of fermentation. Consistent with our results, these molds were the dominant fungal groups in light flavor *Daqu*. *Issatchenkia* is the core yeast in the fermentation process of baijiu. It plays a decisive role in the ethanol production process of fermentation and can produce phenyl ethanol and other metabolites that significantly contribute to baijiu's flavor (Wang, Cai, et al., 2020; Wang, Yang, et al., 2019). *Candida* and *Pichia* play a crucial role in maintaining the stability of the microbial community and fermentation process, resulting in the spontaneous and repeatable baijiu fermentation process. They also enhance the activity of saccharified starch and acid protease during the initial fermentation stage and promote the rate of ethanol synthesis. Furthermore, they improve the aroma of the wine, generating several pleasant aroma compounds, such as ethyl acetate and ethyl butyrate (Wang, Wu, et al., 2017). According to Du et al. (2019), the fungal community in Daqu primarily originates from the Daqu production environment, including fungi such as *Saccharomyces*. Our study results concur with their findings, where *Isatchenkia* and *Pichia* were dominant fungi.

### 3.7 Principal component analysis

PCA analysis is a method of simplifying a large amount of data to obtain preliminary information. It can intuitively reflect the similarities and differences between samples through the distance and symmetry between two points, thereby showing the rationality of sampling and the connections and differences between each sample.

**FIGURE 4:**
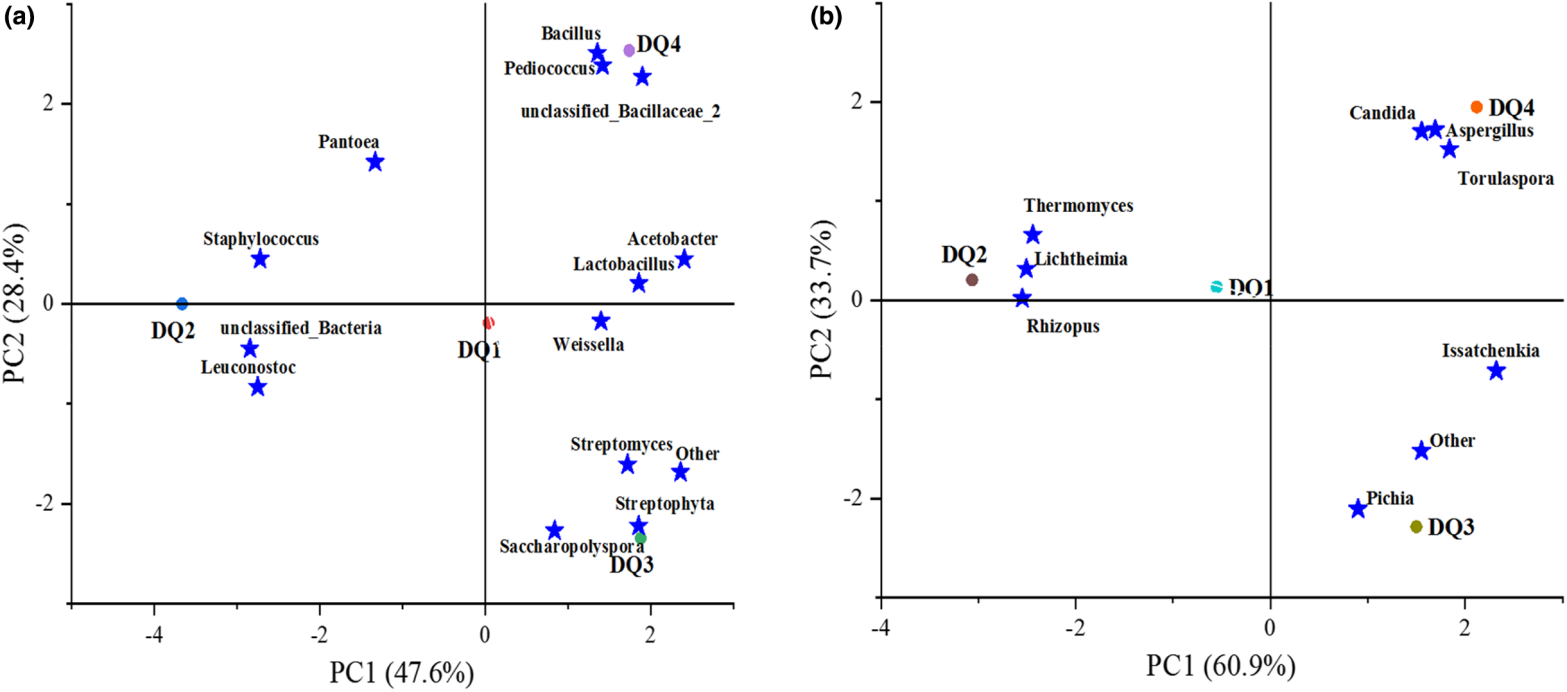
Principal component analysis. (a) Bacteria; (b) fungi.

Figure 4a demonstrated that the principal component represented by the abscissa (PC1) contributed 47.6%, while that represented by the ordinate (PC2) contributed 28.4% to the bacterial flora of *Daqu*. DQ1, DQ2, DQ3, and DQ4 were scattered across different regions, indicating a significant variance in the bacterial flora structure of the four samples. The dominant bacteria in each *Daqu* sample were different. For DQ1, *Acetobacter*, *Lactobacillus*, and *Weissella* were abundant. *Pantoea*, *Staphylococcus*, and *Leuconostoc* accounted for the largest proportion in DQ2 sample. *Streptomyces*, *Streptophyta*, and *Saccharopolyspora* were the dominant bacteria in DQ3, while *Bacillus* and *Pediococcus* were mainly found in DQ4 samples. As shown in Figure 4b, the first principal component contributed to 60.9% of the entire composition, while the second principal component contributed to 33.7%. The four *Daqu* samples were situated in four distinct areas, and their distances from each other were quite substantial, indicating significant differences in the fungi present in *Daqu*. There were significant differences in the dominant fungi of each *Daqu* sample. *Thermomyces*, *Lichtheimia*, and *Rhizopus* were mainly enriched in DQ2 sample, while *Issatchenkia* and *Pichia* are relatively high in DQ3 sample. *Candida*, *Aspergillu*s, and *Torulaspora* were the dominant bacterial groups in DQ4 sample, while there were few dominant fungi in DQ1 sample.

### 3.8 Analysis of volatile components in *Daqu*

As seen from Table 4, 27 volatile compounds were detected from four *Daqu* samples; 25, 26, 25, and 25 substances were detected from DQ1, DQ2, DQ3, and DQ4, respectively. There were 25 substances in common among the four *Daqu*, and DQ2 has the most volatile components. All compounds are divided into four categories: one acid substance, three ketones, three aldehydes, 16 esters, and four alcohols. Esters and alcohols were dominant in the volatile constituents of *Daqu*. The highest ester content in DQ1 was 1931.71 μg/g, while the lowest was 1622.73 μg/g in DQ4. The contents of esters in DQ2 and DQ3 were similar. In addition, the total amount of alcohol substances detected in four kinds of Daqu samples was 2122.78 μg/g, 1117.93 μg/g, 898.1 μg/g, and 690.68 μg/g, respectively. Although the number of alcohols detected is small, the total amount detected is relatively large because alcohols are precursors to synthesizing ester substances. In addition, we found that the content of alcohol substances was positively correlated with the content of ester substances, and DQ1 with more alcohol substances also contained the highest content of ester substances. In contrast, DQ1 contained the least of both alcohol substances and ester substances.

**TABLE 4:** Content of volatile compound in *Daqu* samples.

Volatile compound		CAS No.	Retention index	Description	*Daqu* sample (μg/g)			
					DQ1	DQ2	DQ3	DQ4
Alcohol	N‐amyl alcohol	71‐41‐0	744	Mild bitter	269.01 ± 3.21	191.09 ± 2.34	155.59 ± 1.45	169.44 ± 0.23
	1‐heptanol	111‐70‐6	953	Pungent, slightly bitter	292.16 ± 4.23	46.23 ± 2.67	47.3 ± 1.45	57.5 ± 0.97
	Benzyl alcohol	100‐51‐6	1012	Scorched, slightly bitter	1025.49 ± 2.11	777.73 ± 6.76	550.78 ± 5.34	391.04 ± 5.33
	Isoamyl alcohol	123‐51‐3	736	Cool fragrance	536.12 ± 3.56	102.88 ± 3.32	144.43 ± 2.33	124.75 ± 2.43
Ketone	6‐Methyl‐5‐hepten‐2‐one	110‐93‐0	996	Pungent taste	8.70 ± 2.34	16.68 ± 1.97	16.23 ± 1.12	20.07 ± 1.22
	3‐Octen‐2‐one	18402‐82‐9	1016.4	Nut fragrance	4.35 ± 1.21	10.36 ± 1.11	83.15 ± 2.34	170.35 ± 3.22
	1‐Octen‐3‐one	4312‐99‐6	956	Sweet aroma	1.11 ± 0.67	3.58 ± 0.27	1.35 ± 0.54	1.22 ± 0.23
Ester	Ethyl lactate	97‐64‐3	787	Fragrance	718.78 ± 5.27	957.62 ± 5.89	840.18 ± 6.33	706.66 ± 7.34
	Ethyl acetate	141‐78‐6	612	Fruity odor	932.1 ± 6.76	642.81 ± 7.88	793.46 ± 5.89	680.5 ± 6.87
	Ethyl valerate	539‐82‐2	882	Flowery	13.8 ± 1.11	3.46 ± 0.78	2.22 ± 0.77	1.23 ± 0.02
	Ethyl nonanoate	123‐29‐5	1296	Aroma	81.44 ± 2.43	77.78 ± 2.11	60.63 ± 2.34	79.74 ± 3.21
	Ethyl heptanoate	106‐30‐9	1097	Fruity odor	N.D.	0.22 ± 0.02	N.D.	0.20 ± 0.76
	Ethyl caprylate	106‐32‐1	1180	Fragrance	53.02 ± 1.34	26.68 ± 0.67	17.79 ± 1.32	27.55 ± 1.34
	Ethyl caprate	110‐38‐3	1379	Grassy fragrance	N.D.	0.49 ± 0.03	2.94 ± 0.89	N.D.
	Ethyl caproate	123‐66‐0	982	Fragrance	4.89 ± 0.98	9.71 ± 1.01	5.47 ± 1.07	1.64 ± 0.43
	Ethyl elaidate	6114‐18‐7	2174	Fruity odor	0.81 ± 0.09	4.54 ± 0.88	7.23 ± 1.76	8.83 ± 0.78
	Ethyl linoleate	544‐35‐4	2162	Flowery	73.78 ± 2.76	77.16 ± 3.22	51.03 ± 4.28	66.00 ± 4.87
	Ethyl dodecanoate	106‐33‐2	1595	Cole flower scent	1.63 ± 0.32	2.89 ± 0.56	6.07 ± 0.89	9.34 ± 0.56
	Ethyl stearate	111‐61‐5	2195	Fruity odor	1.32 ± 0.18	2.64 ± 0.32	2.99 ± 0.73	3.41 ± 0.59
	9‐Hexadecenoic acid, ethyl ester	54546‐22‐4	1977	Fragrance	45.89 ± 2.33	43.11 ± 2.22	26.68 ± 1.28	32.74 ± 1.65
	Ethyl myristate	124‐06‐1	1794	Rose scent	0.76 ± 0.02	1.3 ± 0.15	1.24 ± 0.09	1.61 ± 0.64
	Ethyl heptadecanoate	14010‐23‐2	2094	Fragrance	1.92 ± 0.17	2.58 ± 0.35	2.17 ± 0.12	2.71 ± 0.32
	Ethyl 2‐methylbutyrate	7452‐79‐1	849	Fruity odor	1.57 ± 0.24	0.97 ± 0.05	0.46 ± 0.08	0.57 ± 0.11
Aldehyde	Phenylacetaldehyde	122‐78‐1	1045	Fruity odor	14.72 ± 2.22	6.04 ± 0.76	4.6 ± 0.87	5.79 ± 0.56
	Hexanal	66‐25‐1	800	Nut fragrance	10.96 ± 1.45	11.85 ± 1.13	15.17 ± 1.89	20.26 ± 1.32
	Decanal	112‐31‐2	1206	Pungent	33.06 ± 2.56	94.44 ± 3.64	208.27 ± 3.27	397.87 ± 4.76
Acid	N‐caproic acid	142‐62‐1	990	Acidic odor	2.72 ± 0.89	N.D.	N.D.	N.D.

**FIGURE 5:**
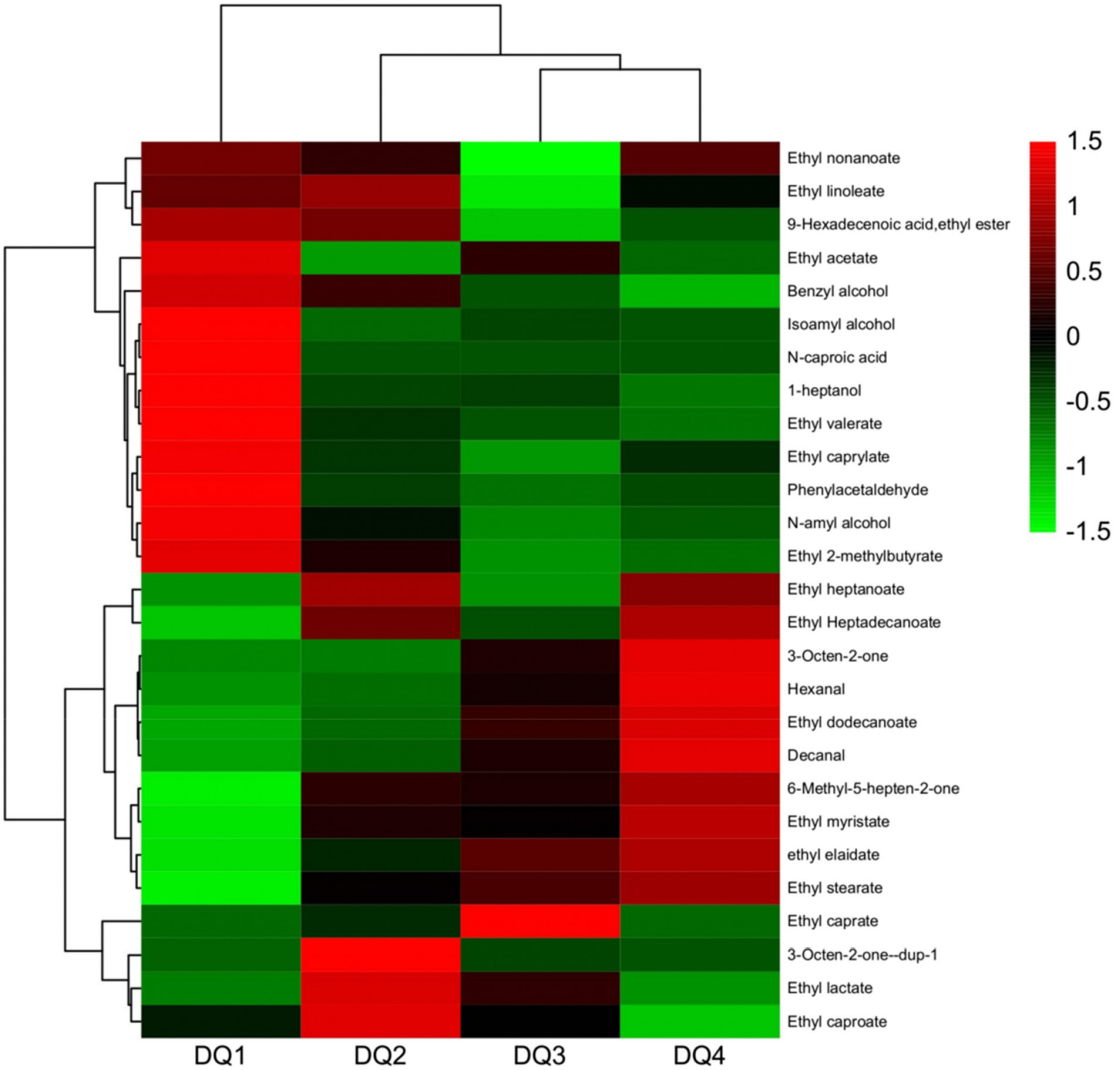
Heatmap of samples' volatile compounds. The color corresponds to normalized mean levels from high (red) to low (green).

The heat map was drawn according to the concentration of flavor substances obtained by a semi‐quantitative method (Figure 5). The dominant flavor substances of unmistakable flavor *Daqu* are mainly concentrated in esters and alcohols. Esters are essential aroma substances among volatile components, and part of the esters in *Daqu* will also enter into the baijiu to form its unique style. The esters in DQ1, DQ2, DQ3, and DQ4 mainly include two types, namely acetate and ethanol esters. Wei et al. (2020) showed that ethanol ester was formed by dehydration and condensation of ethanol and other organic acids under the catalysis of corresponding enzymes. Acetate is synthesized from alcohols except ethanol under catalytic conditions. Many esters were detected in the four *Daqu*, which have a pleasant aroma and play an essential role in the formation of the unique flavor and character of baijiu. Esters and aromatic compounds constitute the unique flavor of *Daqu*, but alcohols are the important material basis for forming esters. Alcohols are produced mainly by yeast using sugars to ferment under aerobic conditions. It can be formed using amino acids under anaerobic conditions or aldehydes to undergo a reduced reaction. The content of higher alcohols can reflect the metabolism of yeast in *Daqu* to a certain extent (Wang, Du, & Xu, 2017). In addition, aldehydes and ketones were also detected in four *Daqu* samples. Microorganisms can degrade aldehydes and ketones to form starch substances in raw materials produced during the glycolysis process, especially under the action of yeast (Lemos et al., 2011). He et al. (2019) found that aldehydes and ketones, together with esters, form the unique fragrance of *Daqu* and that ketones and aldehydes also enter *Daqu* baijiu through fermentation and participate in the formation of aroma as precursor substances of microbial metabolism.

Fatty acids are derived from the metabolism of acid‐producing bacteria and fungi. Fatty acids are also precursors to the formation of esters and, at the same time, produce a specific flavor in baijiu, which affects the taste of baijiu to a large extent. Fatty acid components were detected in DQ2. It was consistent with the previous results showing that DQ2 has the most abundant microbial species. Li et al. (2017) inoculated *Daqu* with Bacillus, Pediococcus, Saccharomycopsis, and Wickerhamomyces using the traditional bio‐intensive inoculation method. After inoculation, the variety of flavor substances in *Daqu* increased, while the fatty acid content also increased significantly, which was consistent with our results. These results indicate that microbial species are closely related to the production of fatty acids.

### 3.9 Analysis of flavor substances

**FIGURE 6:**
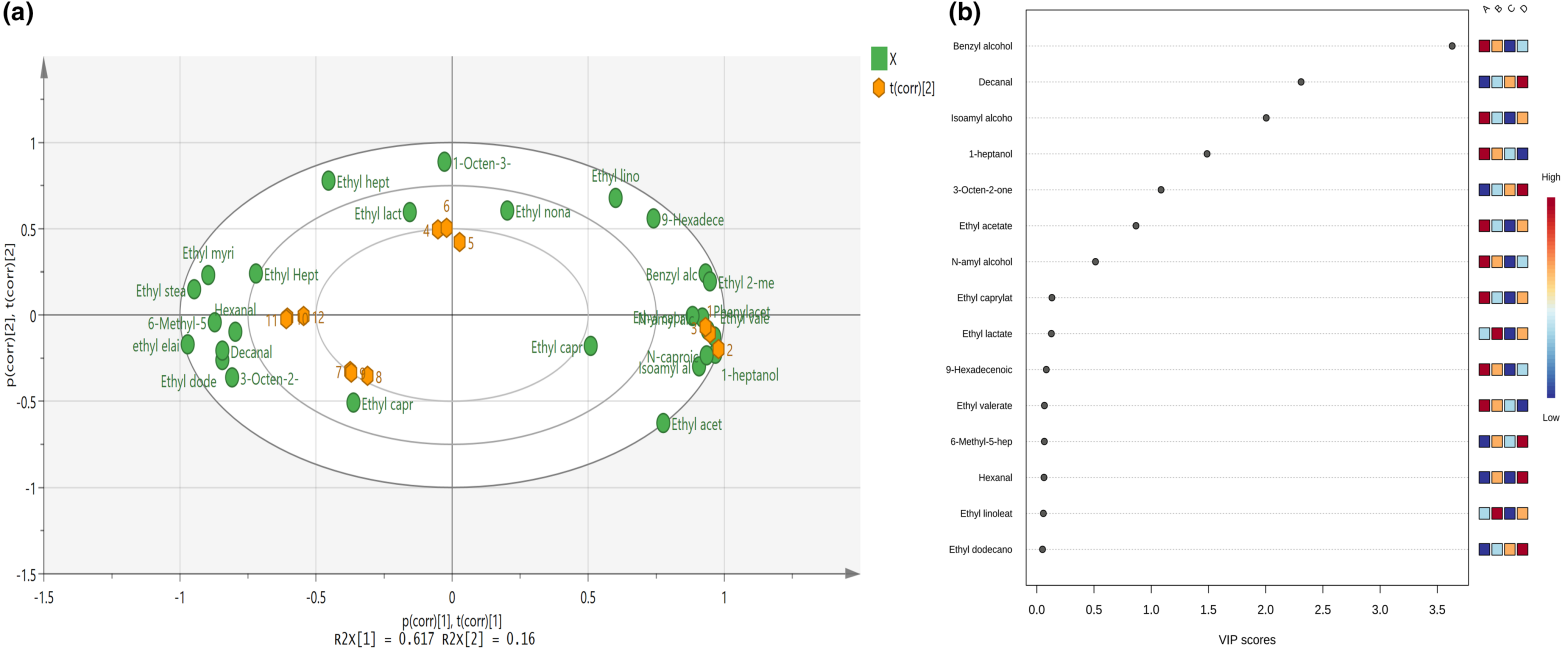
Analysis of the flavor substances. (a) PLS‐DA score graph; (b) VIP graph.

The study employed partial least squares discriminant analysis (PLS‐DA) to evaluate the discrepancies in flavor metabolites among four Daqu samples. The scatter plot depicted in Figure 6a using PLS‐DA revealed that there were substantial variations in the volatile components among the four samples, indicating a significant difference in the metabolite compositions of Daqu in various regions. The primary flavor substances were ethyl acetate and ethyl lactate. Based on the variable importance for the projection (VIP), a total of 15 metabolites were identified to have a considerable contribution to the metabolic composition differences in this model, as illustrated in Figure 6b.

The yeast in *Daqu* is closely related to the formation of ethyl acetate. The ethyl acetate content in baijiu can be increased by screening the yeast strains with a high yield of ethyl acetate. The critical precursor of Ethyl lactate is lactic acid, an important product of LAB. Therefore, LAB in *Daqu* positively affected the formation and accumulation of Ethyl lactate. Ethyl acetate and ethyl lactate were the prominent esters in four light‐flavor *Daqu*, this is consistent with the report of Zheng et al. (2012), but the ethyl acetate and ethyl lactate were not the different main substances in four *Daqu* (VIP <1). VIP represents the contribution of volatile components to the sample to screen out important characteristic substances. It is generally believed that VIP >1 indicates that this variable plays an important role, and the larger the VIP value, the more significant the difference between samples. Benzyl alcohol, isoamyl alcohol, and other alcohols were the different main substances of the four *Daqu* samples (VIP >1), consistent with the microbial community structure analysis results. Yeast played a leading role in the production of alcohol. The abundance of Issatchenkia, Pichia, and Candida varied greatly, leading to significant differences in the contents of alcohol (Figures 3 and Figure 6).

## 4 CONCLUSION

The study utilized four Daqu samples from various regions of Shanxi Province to evaluate their physical and chemical properties. The microbial community structure and volatile components were examined using NGS and HS‐SPME–GC–MS. The results showed that there were no notable variations in water content and esterase activity among the four *Daqu* samples, but significant differences existed in acidity, protease, and saccharifying enzyme activity. Specifically, DQ2 displayed the highest saccharifying enzyme activity and acidity value, while DQ4 had the highest protease activity. The results of microbial diversity analysis showed significant differences in the dominant bacteria and fungi among the four *Daqu* samples. *Lactobacillus* and *Weissella* were the highest in the DQ1 sample, while *Leuconostoc*, *Streptomyces*, *Streptomyces*, *Rhizopus*, and *Lichtheimia* were the highest in the DQ2 sample. The abundance of *Streptococcus*, *Staphylococcus*, *Saccharopolyspora*, *Isatchenkia*, and *Pichia* is the highest in the DQ3 samples. The abundance of *Bacillus*, *Pediococcus*, *Acetobacter*, *Torulaspora*, *Aspergillus*, and *Candida* was the highest in the DQ4 sample. Among the 27 common volatile substances detected in *Daqu*, there are the most esters, with ethyl acetate and ethyl lactate being the most abundant, followed by a large amount of alcohol. There is no significant difference in the types of volatile components among the four *Daqu*. However, there are significant differences in the content of each substance, which may be related to differences in microbial communities. Therefore, in actual production, four kinds of *Daqu* can be mixed in a particular proportion to improve the baijiu yield and obtain baijiu with good flavor. It will also provide a theoretical basis for further optimizing the proportion of mixed *Daqu*. However, this paper only collected *Daqu* from four representative cities as the research object. In the future, we will collect a large number of *Daqu* from other regions, so as to provide a theoretical basis for a comprehensive analysis of the nature of Shanxi light flavor *Daqu*.

## AUTHOR CONTRIBUTIONS

**Panpan Hu:** Data curation (equal); investigation (equal); methodology (equal). **Ji Wang:** Formal analysis (equal); resources (equal); visualization (equal). **Urooj Ali:** Investigation (equal); validation (equal); writing – original draft (equal). **Tariq Aziz:** Funding acquisition (equal); project administration (equal); writing – review and editing (equal). **Manal Y. Sameeh:** Formal analysis (equal); methodology (equal); validation (equal). **Caiping Feng:** Supervision (equal); writing – review and editing (equal).

## FUNDING INFORMATION

The funding for this research was provided by two sources: The Lyuliang high‐level scientific and technological talents critical research and development project (Grant No. 2022RC19) and the Shanxi Province universities teaching reform and innovation project (Grant No. J20221148).

## CONFLICT OF INTEREST STATEMENT

The authors declare no conflict of interest.

## Data Availability

The data presented in this study are available in the article.
